# Differential effects of threat types on attentional processes: a comparison of snakes and blood-injury-injection stimuli

**DOI:** 10.3389/fpsyt.2024.1498709

**Published:** 2024-12-24

**Authors:** Andras Norbert Zsido, Botond László Kiss

**Affiliations:** ^1^ Institute of Psychology, Faculty of Humanities and Social Sciences, University of Pécs, Pécs, Hungary; ^2^ Contemporary Challenges Research Centre, University of Pécs, Pécs, Hungary; ^3^ Szentágothai Research Centre, University of Pécs, Pécs, Hungary

**Keywords:** attentional biases, orienting, executive attention, number matrix, anxiety, disgust sensitivity, emotion regulation

## Abstract

**Introduction:**

Previous research on the visual processing of threats has largely overlooked the Q8 distinct effects of various types of threats, despite evidence suggesting unique brain activation patterns for specific fears. Our study examines the differential effects of threat types on attentional processes, focusing on snakes and blood-injury-injection (BII) stimuli. We sought to test whether these two types of threat stimuli, as taskirrelevant distractors, would lead to similar effects in a visual search task.

**Methods:**

Participants were exposed to emotionally charged stimuli of varying arousal (medium and high) and neutral pictures of low arousal as task-irrelevant distractors while performing a primary visual search task.

**Results:**

We found that BII-related distractor pictures interfered with attention to the primary task, resulting in slower reaction times compared to snake pictures. In addition, BII-related medium arousal stimuli decreased, but high arousal facilitated both orienting and executive attentional performance. Exploratory analysis of personality traits revealed differential effects of trait anxiety and disgust sensitivity, highlighting the unique mechanisms underlying fear responses. In addition, participants who used adaptive emotion regulation strategies showed better performance in overcoming the interference of threat stimuli on attention.

**Discussion:**

These findings underscore the importance of considering individual differences and emotion regulation strategies in fear research and provide insight into the complex interplay between threat perception and attentional processes.

## 1 Introduction

It has long been postulated that threat is prioritized in visual attentional processing (1–6). For example, threatening targets are found faster than neutral ones (7–9), and threatening distractors are harder to ignore (10–13). One possible explanation for this is that the emotional arousal elicited by such stimuli is processed quickly, marking these objects for closer inspection (14, 15). Arousal theories (16–19) argue that threats elicit a state of heightened physiological activation (generalized arousal) that enhances processing in the survival circuitry and, consequently, increases the capacity of working memory. For task-relevant threats, behavioral effects include faster search times, increased accuracy, and more frequent fixations compared to neutral stimuli. In contrast, when threats are used as task-irrelevant distractors, the arousal effect may emerge in a less straightforward manner. This is best described by the arousal stimulation effect (20, 21), which posits that the threat distractor initially attracts attention and reduces performance on the primary task. However, this can later be overcome if the arousal level of the stimulus is high, resulting in a heightened state of physiological activation and thus facilitated overall attentional performance. Although theories describing threat detection and defensive behaviors are committed to a universal explanation that applies to all types of threats (e.g., animate, objects, injuries, social), the generalizability of these findings across different types of threats has not yet been addressed.

Several previous studies have shown that different types of threats can elicit unique responses (22), including specific patterns of brain activity. For example, when people with snake phobia (a subtype of animal phobia) or dental phobia (a subtype of blood-injection-injury [BII] phobia) are shown phobia-relevant threatening videos in an fMRI scanner, there is a discrepancy in the regions of the brain that are activated (23). For snakes, the insula, anterior cingulate cortex, and thalamus showed increased activity, whereas for dental threats, the prefrontal and orbitofrontal cortices were more active. Similarly, another study (24) showing pictures of spiders to people with spider phobia (again, a subtype of animal phobia) and pictures of blood, injection, and injuries to people with BII phobia found activation in the anterior cingulate and insula for spiders and activation in the occipito-temporo-parietal regions and thalamus for BII images. This study also found that anxiety and disgust sensitivity were associated with activation in different regions in the two subtypes. Similarly, results from studies comparing brain activity correlates of stimulus exposure between phobic patient groups may indicate differences in emotion regulation processes (25). Emotion regulation, which involves strategies for modulating emotional responses, is crucial in determining how individuals cope with and process threatening stimuli. Beyond the findings on high anxiety, there is evidence that specific emotion regulation strategies, such as reappraisal or suppression, may differentially influence attentional biases and threat-related behaviors (26). In addition, the nature of the threat itself may elicit different regulatory demands: animal-related fears may elicit rapid, automatic regulatory mechanisms due to their evolutionary salience, whereas BII-related fears may elicit more complex regulation associated with disgust and fainting responses (27). These findings, along with survey-based studies (28, 29), may suggest that both threat processing and emotion regulation may differ for discrete types of threat (such as those related to animals or BII).

Although the concept of unique background mechanisms for different types of threats is not novel, studies have not addressed whether this translates into differences in behavioral responses to these threats. A lack of connection between clinically oriented phobia studies and cognitive experimental studies focusing on threat may account for this gap. In phobia research, for example, studies (30–32) over 30 years ago showed that some phobias are better described by a predator-defense model (fear-relevant), while others fit better with a disease-avoidance model (disgust-relevant). The predator-defense model can be applied to threatening objects and situations that are perceived as predatory and are likely to cause attack or immediate physical harm. In contrast, the fear response in the disease-avoidance model is driven by disgust and contamination sensitivity associated with threatening objects and situations that may spread disease, cause illness, or elicit physiological manifestations of the food rejection response (e.g., nausea). Recent clinically oriented studies point to the diversity of key elements of different threats and argue that the defensive (behavioral) response to a threat depends on what triggers the fear response (27, 33). For example, while disgust is less associated with animals that are capable of (seriously) injuring humans, such as snakes, visual features (e.g., body or head shape) are an important factor in determining behavior. In fact, visual features alone are capable of triggering the fear response through a subcortical pathway (34, 35). Not surprisingly, a large number of previous studies focusing on the attentional prioritization of threats have used animate stimuli, particularly snakes (36–38). In contrast, disgust is an important factor in the evaluation of BII-related stimuli, while visual features play a lesser role (27, 39). There has been little research on the presence of attentional prioritization of BII-related threats (40). This calls for a systematic investigation of attentional prioritization of threats to see if it can be applied similarly to all types of threats.

The aim of the current study was to test if various types of threatening stimuli (i.e., snakes and BII-related images) as task-irrelevant distractors would lead to similar effects in a visual search task. We sought to investigate if snakes and BII-images would capture and hold the attention of participants. Further, we aimed to test if the arousal stimulation effect applies similarly to both types of threats. That is, we expected that performance will be lower for medium arousal threats compared to neutral (low arousal) images, while high arousal threats will compensate for this effect resulting in a performance similar to neutral images.

Because people differ in their behavior, we further explored interactions with personality traits. Previous studies have shown that the effect of threats on attention would be more pronounced in people with higher (compared to lower) levels of trait anxiety (41, 42). Thus, in the present study, we expected that higher levels of trait anxiety would impair performance on the visual search task. Similarly, people who are more afraid of a stimulus have been shown to be more distractible by the presence of the feared object (43–45). This also fits well with motivational relevance and appraisal theories suggesting that stimuli that are perceived to be motivationally or personally relevant are more likely to attract and hold attention (45, 46). Thus, we expected that people reporting higher levels of BII fear would decrease performance on BII trials, whereas higher levels of snake fear would decrease performance on snake trials. Adaptive emotion regulation strategies may help people cope with the negative emotions that threats elicit (47). In contrast, maladaptive emotion regulation strategies tend to increase negative emotions (48). Thus, we expected that more frequent use of adaptive emotion regulation strategies would help people focus on the primary task and improve performance. In contrast, maladaptive emotion regulation strategies will lead to decreased performance. Finally, disgust sensitivity is associated with greater avoidance of stimuli that are perceived as aversive (26). Thus, higher levels of disgust will lead to increased performance because the avoidance of negative stimuli will instead lead to a focus on the task.

## 2 Materials and methods

### 2.1 Participants

We conducted an *a priori* power analysis using G*Power (49). The analysis, based on previous studies (21, 50, 51), indicated that the required minimum total sample size (f= 0.25, 1-β= 0.95, r=0.5) was 28. We collected data from 30 volunteers (11 males, 19 females) who were undergraduate students at the university in which the data were collected. Their mean age was 22.4 (SD=4.14). All participants identified as Caucasian. See **Table 1** for the descriptive results regarding fear levels of participants.

**Table 1:** Descriptive statistics and reliability values for the questionnaire scores.

	Trait anxiety	Disgust sensitivity	Adaptive ER	Maladaptive ER	BII fear	Snake fear
Mean	11.2	38.5	32.3	20.6	20.6	2.82
Median	11.0	36.0	34.5	21.0	20.0	3.00
SD	3.41	9.74	6.58	5.34	6.86	2.25
McDonald’s ω	.838	.868	.808	.798	.891	z.721

All participants were right-handed and reported normal or corrected-to-normal vision. Our research was approved by the Hungarian United Ethical Review Committee for Research in Psychology and was carried out in accordance with the Code of Ethics of the World Medical Association (Declaration of Helsinki). All participants provided written informed consent.

### 2.2 Stimuli

**Figure 1:**
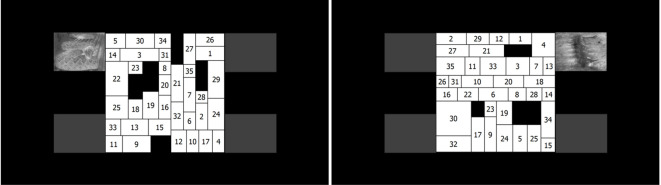
The visual search task and layout of the stimuli used in the present study. The left panel shows a snake image with high arousal, the right panel shows a Blood-Injury-Injection-related image with medium arousal appearing beside the corner of the matrix. Please note that while we added gray rectangles to demonstrate the four possible positions an image could appear in, these were not used during the experiment.

**Figure 1** shows exemplars of the final stimuli set. The visual search task consisted of searching for numbers (sequentially) in matrices that were created using a special matrix generator program. The size of the matrices was 700x700 pixels (17.55° x 17.55°). Each matrix contained 35 white rectangles (with numbers) – the width and height of these varied from 150 to 300 pixels (visual angle of 3.79° to 7.57°) – and 3 to 6 black ones (without numbers). The black rectangles were randomly distributed among the white ones and were added simply to maintain the same overall global shape in the search area. The width and height of the black rectangles varied from 70 to 230 pixels (visual angle of 1.51° to 4.98°). Both the matrices and rectangles within had a 2-pt black border drawn around them. The numbers ranged from 1 to 35 and were randomly distributed among the rectangles. Each number appeared only once in a given matrix. All the rectangles contained a number printed in black in 32-pt Tahoma font. In each trial, a number matrix was superimposed on a facial image. The matrix generator program is freely accessible from http://baratharon.web.elte.hu/nummatrix/.

Previous studies (21, 50, 52) using the number matrix task have identified two key measures: finding the first number (i.e., 1) and search time (finding numbers 1 through 10). Finding the first number is a simple visual search task in which participants search for a target among distractors guided by the features of that target, and therefore primarily requires attentional orienting (53, 54) because it relies more on bottom-up rather than top-down processing, as the primary determinant of behavior is the difference in appearance between the number one and the other distractor numbers in the array. In contrast, the task of searching through the number matrix relies more on top-down rather than bottom-up processes because searching through the remaining numbers requires participants to control their attention and constantly maintain their search target, updating the information in WM each time a new target digit is found (55, 56).

For each trial, a task-irrelevant picture appeared in one of the four possible spatial positions; i.e., each of the four corners of the screen 12.5° from the center of the matrix. Both snakes and BII-related images were taken from previously validated databases (39, 57–59). We used the arousal ratings described by these studies. We selected 16 from each category so that the image shown on each trial was unique. The arousal of each image was determined on 9-point Likert-type scales; snake and BII-related images did not differ in terms of arousal (*t*<1, *p*>0.1). We also defined "medium" (ratings between 5 and 7) and "high" (ratings above 7) arousal categories; images in the medium and high arousal categories were significantly different in terms of arousal (*t*>2, *p*<0.01). We also used neutral images as controls – sourced from Internet searches – with low arousal that had a similar content (e.g., a healthy non-injured hand for the BII category or a caterpillar for the snake category) but were nonthreatening. This category also significantly differed from the medium and high arousal categories (*F*>2, *p*<0.01). First, we resized the images to the same size (300 x 225 pixels, visual angle of 7.57° to 5.68°). Then, we equated the pictures on low-level perceptual features (including brightness, contrast, spatial frequency) using the SHINE Matlab toolbox (60). As a result of this process the images were converted to grayscale. The images did not differ in visual complexity based on log JPG file size measures (61, 62).

### 2.3 Questionnaires

Personality-related factors were measured with questionnaires. See **Table 1** for descriptive statistics and internal consistency scores. We measured *anxiety* with the short, five item version of the Spielberger State-Trait Anxiety Inventory (63). Higher scores indicate higher trait anxiety levels. We measured *disgust* sensitivity using the Revised Disgust Scale (64). Individuals with higher scores are more likely to experience disgust and are more sensitive to disgust-inducing content. We used the 18-item version of the Cognitive Emotion Regulation Questionnaire (65) to assess the participants' putatively *adaptive* emotion regulation strategies and putatively *maladaptive emotion regulation strategies*. The higher the score of individual subscales the more that specific cognitive strategy is used. Further, we measured *fear of snakes* using the short Snake Questionnaire (66) and we assessed *BII-related fears* with the short version of the Medical Fear Survey (67, 68). A higher score indicates higher levels of fear for both questionnaires.

### 2.4 Apparatus and procedure

Participants were engaged in the study in small groups on up to 8 computers simultaneously (with identical hardware and software profiles) in a computer room. Participants were seated in separated workstation booths, at approx. 60 cm in front of 21.5-inch LCD monitors with a resolution of 1920 × 1080, 16:9 aspect ratio, a refresh rate of 60 Hz, and a color depth of 16.7 M. Stimuli were presented and randomized using PsychoPy v3.0 (69). Experimental sessions were monitored by one research assistant. Participants started the task after being given detailed verbal and written instructions, as well as an opportunity to ask any questions of clarification. Participants filled in all the questionnaires before starting the experiment.

The experiment began with two practice trials (with scattered images as distractors). We did not analyze these trials. This was followed by 48 experimental trials. All participants completed every trial (with neutral, snake, and BII-related images) regardless of questionnaire scores. Each trial started with a white fixation cross presented for 1000 ms on a black background. Then, the number matrix appeared in the center of the screen simultaneously with an image in one of the four possible positions; the background was black. Participants’ task was to locate the numbers in ascending order starting with the number one and to indicate each find by clicking on the numbers using the computer mouse. We instructed participants to try and complete the task as quickly and accurately as possible. Each new trial was initiated by the participant by pressing the spacebar, therefore they had the opportunity to rest or take a break if they felt it necessary. The number matrices used were randomized across participants and trials. One session of data collection lasted approximately 60 to 90 min.

## 3 Results

### 3.1 Analytic approach

We identified and removed outlier trials, defined as those more than 2.5 standard deviations longer than the sample mean (less than 1% of trials). Two participants were excluded from our analyses for having mean RTs > 2.5 standard deviations above the sample mean. The final sample size that was used in the analyses was 28.

Then, we performed two 2x3 ANOVAs to test the effects of distractor Stimuli Type (BII, snake) and Arousal (low, medium, high) on performance. Our behavioral measures of performance included examining RTs (in s) for the time needed to find the Number 1, and the total search time (in s) for finding Numbers 1 through 10. All assumptions were met for the ANOVAs. Statistical results are presented in tables instead of in text to make the description of the results easier to follow.

Finally, we used General Linear Modelling to test what measured personality-related variables were significant predictors of the behavioral measures of performance. GLM is conceptually equivalent to ANCOVA, as it allows us to include multiple predictors in the same model while accounting for covariates. We tested four models, with finding the Number 1 and the total search time as dependent variables separately for BII and snake-related conditions. In all models, the independent predictors were self-reported ratings of anxiety, disgust, fear (BII or snake based on the condition), maladaptive and adaptive emotion regulation strategy. All assumptions were met for the GLMs.

### 3.2 Finding the first number

**Figure 2:**
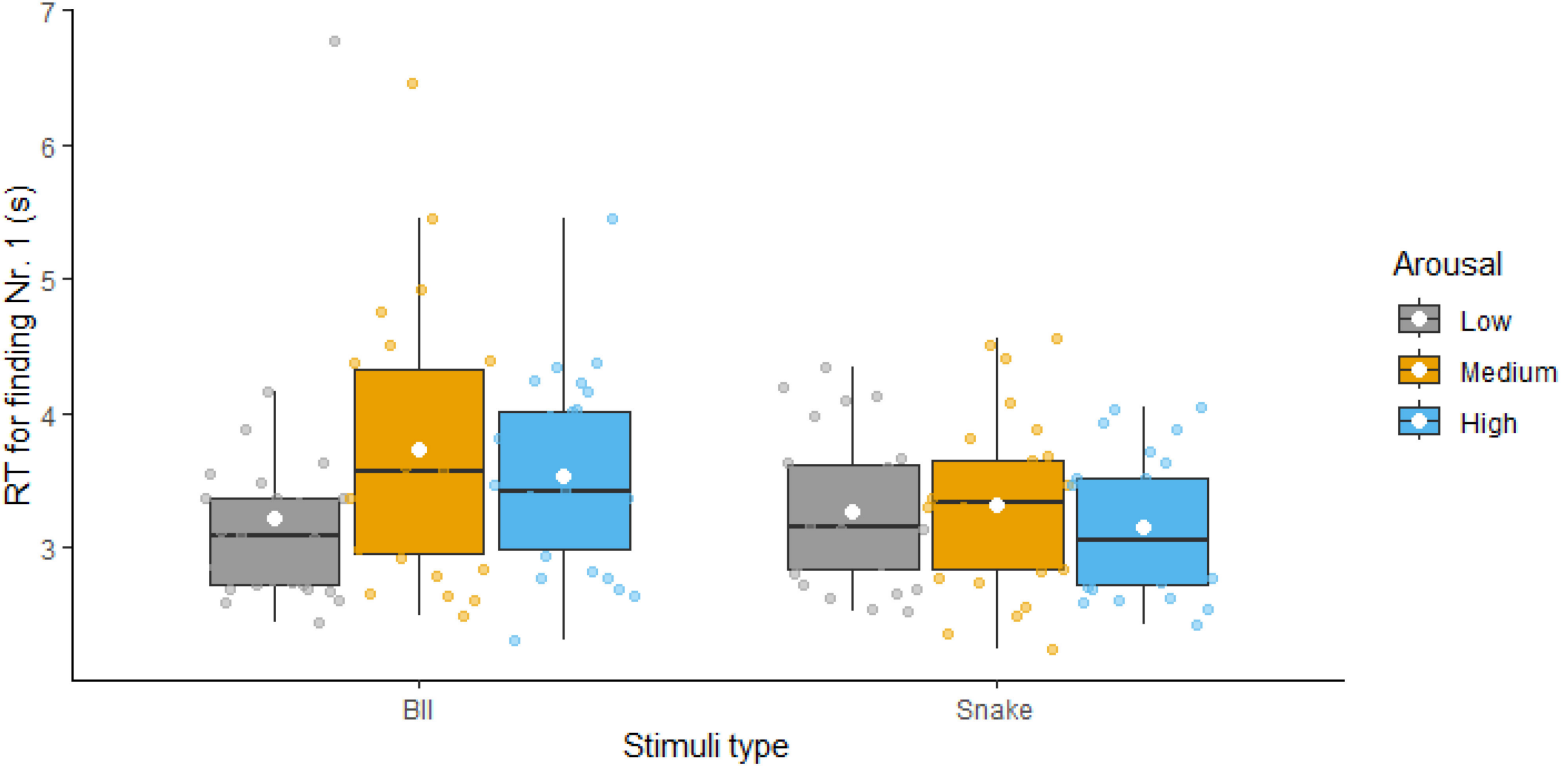
Reaction times for finding the first number with low, medium and high arousal distractor images visualized as boxplots (separately for Blood-Injection-Injury and Snake-related threatening images). White diamonds show the means of the variables.

We began by examining RTs for finding the first number to test our prediction that the arousal stimulation effect would be similar to both animate and BII-related threatening stimuli. **Figure 2** presents the descriptive statistics for these comparisons; statistical results are presented in **Table 2**. The ANOVA revealed a significant main effect of type – with BII-related images being more distractive, thus resulting in higher RTs compared to snake-related images – and a significant main effect of arousal – replicating the arousal stimulation effect. However, the interaction between the two factors was also significant. Follow-up analyses revealed that the arousal effect was only significant for BII-related images (not for snake-related ones) and that both medium and high arousal stimuli distracted participants. Thus, while we found evidence for an arousal effect, contrary to our predictions, performance in terms of RTs was different for snakes compared to BII-related stimuli.

**Table 2:** Detailed statistical results for finding the first number with main effects, interactions and follow-up tests.

		df	F/t	p	η²p	Mean difference
Type		1, 26	10.93	0.003	0.296	
Arousal		2, 52	6.12	0.004	0.190	
	Low - Medium	26	-3.31	0.007		-0.315
	Low - High	26	-2.18	0.093		-0.147
	Medium - High	26	1.62	0.257		0.168
Type x Arousal		2, 52	12.94	< .001	0.332	
BII		2, 52	11.6	<.001	0.309	
	Low - Medium	26	-4.74	< .001		-0.635
	Low - High	26	-4.17	< .001		-0.450
	Medium - High	26	1.16	0.488		0.185
Snake		2, 52	1.99	0.147	0.071	

P-values for pairwise comparisons are Tukey-corrected values.

### 3.3 Total search time

**Figure 3:**
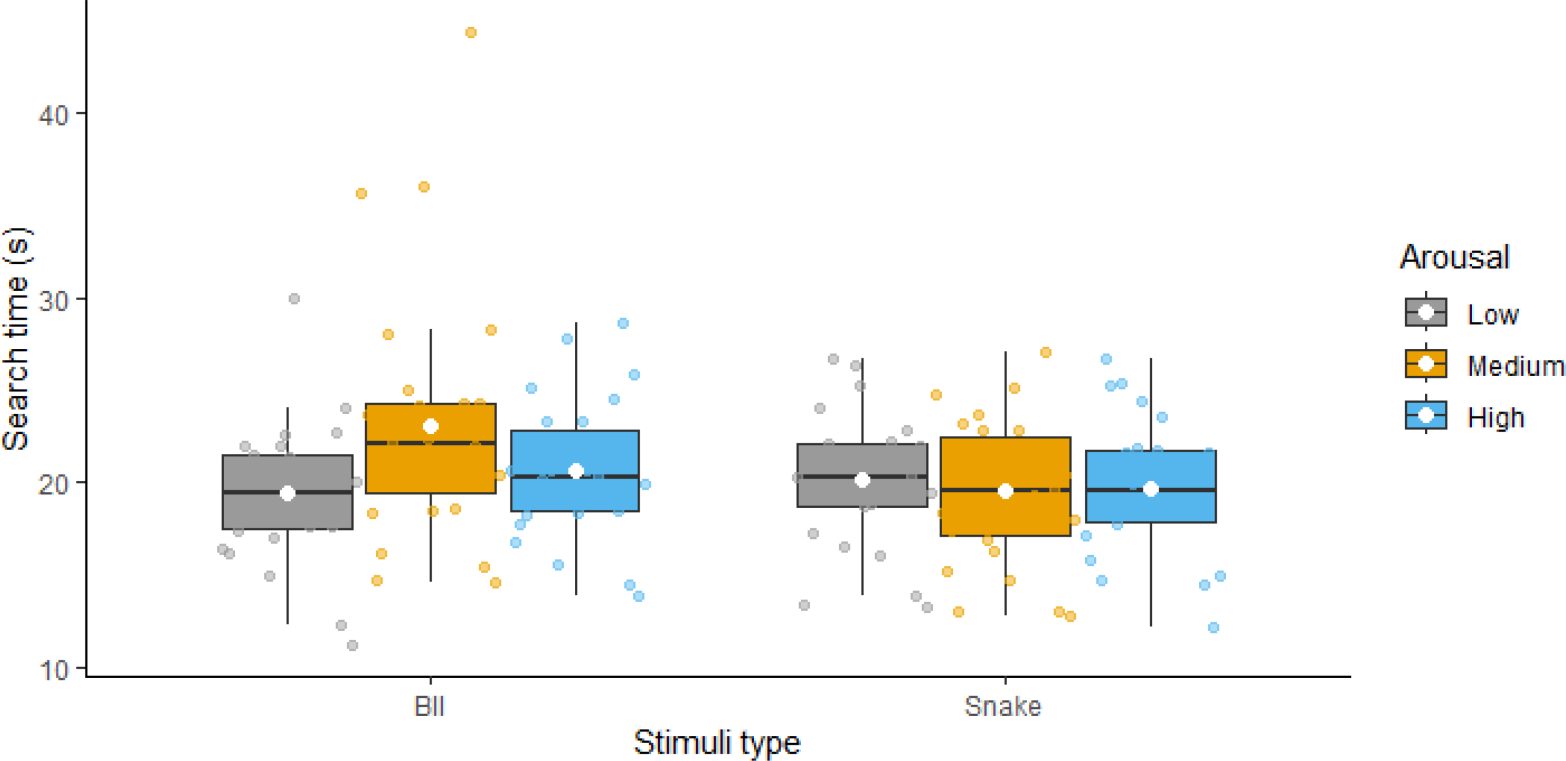
Reaction times for finding all the numbers with low, medium and high arousal distractor images visualized as boxplots (separately for Blood-Injection-Injury and Snake-related threatening images). White diamonds show the means of the variables.

We then examined search times for finding numbers 1 through 10 to test our prediction regarding the arousal stimulation effect. **Figure 3** presents the descriptive statistics for these comparisons; statistical results are presented in **Table 3**. The ANOVA revealed a significant main effect of type with BII-related images being more distractive, thus resulting in higher RTs compared to snake-related images. The main effect of arousal was nonsignificant. However, the interaction between the two factors was significant. Follow-up analyses showed a significant arousal effect for BII-related images but no significant effect of arousal for snake-related images. Thus, while we found evidence for an arousal effect, contrary to our predictions, this was only true for BII-related but snake-related stimuli.

**Table 3:** Detailed statistical results for finding all the number with main effects, interactions and follow-up tests.

		df	F/t	p	η²p	Mean difference
Type		1, 24	5.31	0.030	0.181	
Arousal		2, 48	1.64	0.205	0.064	
Type x Arousal		2, 48	5.52	0.007	0.187	
BII		2, 50	6.19	0.004	0.199	
	Low - Medium	25	-2.68	0.033		-2.41
	Low - High	25	-2.09	0.112		-1.09
	Medium - High	25	2.30	0.075		1.32
Snake		2, 48	1.61	0.210	0.063	

The detailed descriptive statistics for the behavioral variables included in the statistical analysis are presented in **Table 4**.

**Table 4:** Descriptive data regarding the reaction times for finding the first number (Finding Nr 1) and for finding Numbers 1 through 10 (Search).

Measure	Type	Arousal level	Mean (s)	95% CI	
				Lower	Upper
Finding Nr 1	Snake	Low	3.27	3.06	3.49
		Medium	3.27	3.04	3.50
		High	3.12	2.93	3.31
	BII	Low	3.09	2.92	3.26
		Medium	3.73	3.35	4.11
		High	3.54	3.27	3.82
Search time	Snake	Low	20.1	18.6	21.6
		Medium	19.0	17.5	20.5
		High	19.6	18.1	21.2
	BII	Low	19.4	17.9	21.0
		Medium	21.9	20.0	23.9
		High	20.5	18.9	22.0

The reaction times are presented in each picture type (Snake or BII) and with every arousal level (low, medium, and high).

### 3.4 Effect of personality-related factors

**Figure 4:**
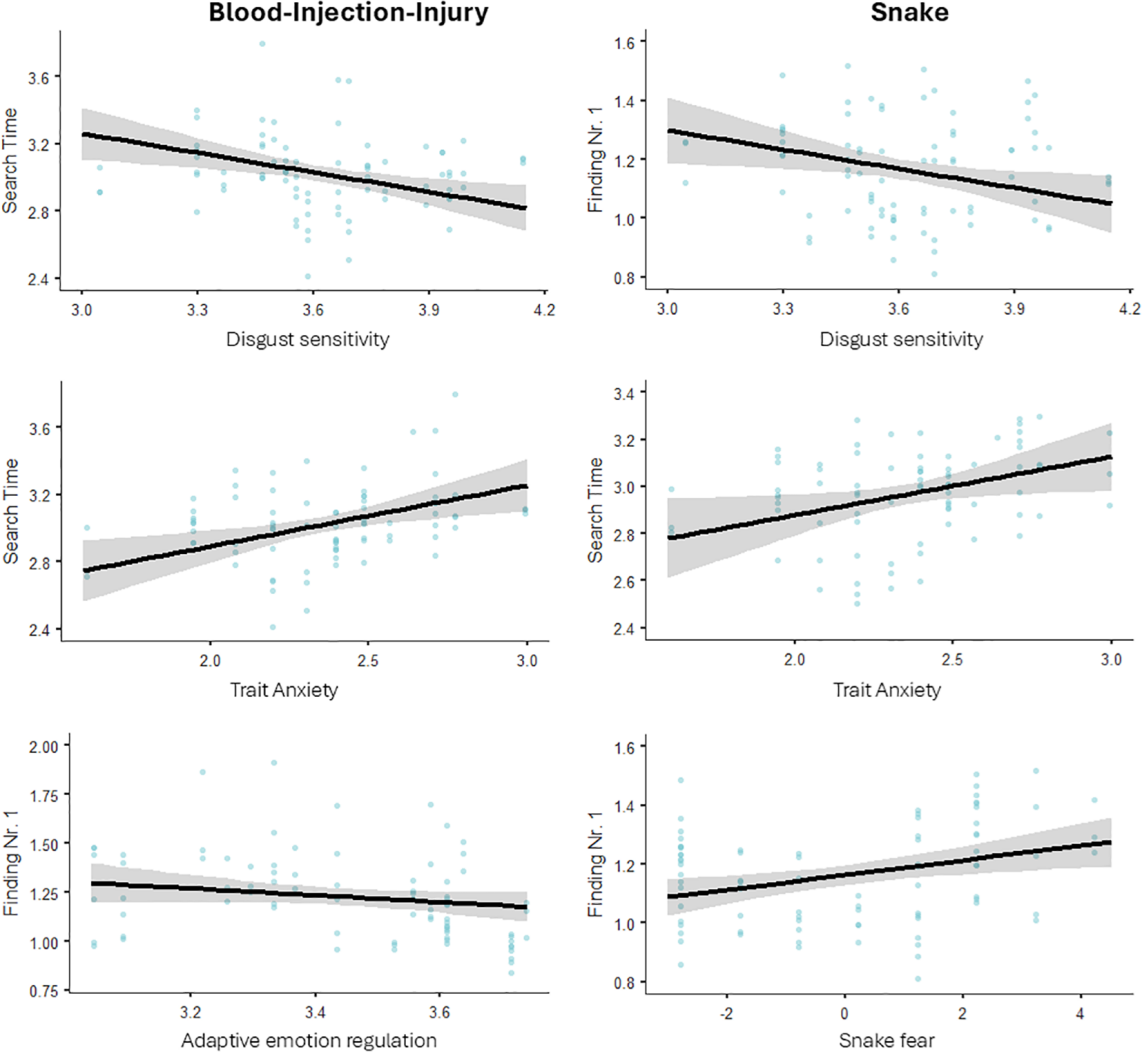
The relationship between personality traits and search performance.

Finally, we tested if individual differences in threat-related personality factors have an effect on RTs for finding the first number and search times for finding numbers 1 through 10. **Figure 4** presents the descriptive statistics for the significant effects; statistical results are presented in **Table 5**.

**Table 5:** Results of the General Linear Models with search performance as dependent variables and personality traits as independent predictors.

Type	Measure	Effect	df	F	p	η²p	β
BII	Finding NR 1	Model	5, 76	3.331	0.009	0.180	
		Trait anxiety	1, 76	1.144	0.288	0.015	0.18096
		Disgust sensitivity	1, 76	1.695	0.197	0.022	-0.19131
		*Adaptive ER*	*1, 76*	*8.665*	*0.004*	*0.102*	*-0.32870*
		Maladaptive ER	1, 76	<0.001	0.990	0.000	-0.00214
		Fear (BII)	1, 76	0.311	0.579	0.004	0.06899
	Search time	Model	5, 75	4.054	0.003	0.213	
		*Trait anxiety*	*1, 75*	*7.657*	*0.007*	*0.093*	*0.4665*
		*Disgust* sensitivity	*1, 75*	*8.944*	*0.004*	*0.107*	*-0.4449*
		Adaptive ER	1, 75	0.751	0.389	0.010	0.0967
		Maladaptive ER	1, 75	0.522	0.472	0.007	0.1219
		Fear (BII)	1, 75	0.179	0.674	0.002	0.0517
Snake	Finding NR 1	Model	5, 77	4.270	0.002	0.217	
		*Trait anxiety*	1, 77	2.518	0.117	0.032	0.272
		*Disgust* sensitivity	*1, 77*	*4.362*	*0.040*	*0.054*	*-0.280*
		Adaptive ER	1, 77	1.244	0.268	0.016	-0.127
		Maladaptive ER	1, 77	0.524	0.471	0.007	0.121
		*Fear (snake)*	*1, 77*	*6.892*	*0.010*	*0.082*	*0.291*
	Search time	Model	5, 73	2.124	0.072	0.127	
		*Trait anxiety*	*1, 73*	*5.266*	*0.025*	*0.067*	*0.4344*
		Disgust sensitivity	1, 73	0.227	0.636	0.003	-0.0735
		Adaptive ER	1, 73	0.875	0.353	0.012	0.1206
		Maladaptive ER	1, 73	0.114	0.737	0.002	-0.0606
		Fear (snake)	1, 73	1.960	0.166	0.026	0.1651

The italized values indicate significant results.

For BII-related stimuli, the linear models revealed a significant negative effect of adaptive ERs on RTs finding number 1; and a significant positive effect of anxiety and a negative effect of disgust sensitivity on search times. That is, participants using more adaptive ERs were less distractible by BII-related images initially. Further, being more anxious increased search times while being more prone to experience disgust decreased search times.

For snake stimuli, the linear models revealed a significant negative effect of disgust sensitivity and a positive effect of fear on RTs finding number 1; and a significant positive effect of anxiety on search times. That is, being more prone to experience disgust decreased, while being more fearful increased distractibility by snake images initially. Further, being more anxious increased search times.

## 4 Discussion

It has long been argued that threats lead to attentional biases, such as faster detection or greater distraction (1, 2, 11–13, 34). However, to our knowledge, no previous study has tested whether these biases are universally present across threat types. This is particularly intriguing in light of neuroimaging studies showing distinct patterns of brain activation for threats associated with different fears (23, 24). An (re-)emerging concept in phobia research is that not all fears are driven by the same underlying mechanisms. Therefore, in the present study, we sought to compare threat types (snake vs. BII-related) in a well-established attentional task to investigate whether these threats, as task-irrelevant distractors, elicit similar behavioral responses. We found that only BII-related images interfered with attention to the primary task. When such an image was presented, participants were slower than when a snake image was present. Furthermore, we found evidence for the arousal stimulation effect only for BII images. That is, these images distracted participants, resulting in decreased task performance, but this was overcome when the arousal level of the stimulus was high. Based on previous studies (70–72) that measured physiological correlates of the dynamics of subjective emotional arousal while viewing different emotional content, we suggest that the high stimulus arousal resulted in a heightened state of physiological activation, thereby facilitating overall attentional performance. These results may provide further support for the concept of unique background mechanisms for specific types of threat. Based on our results, this also translates into differences in behavioral responses to these threats.

The time to find the first number was slower when the task-irrelevant image was a BII-related threat than when it was a neutral or animal-related threat. The result for BII-related stimuli is consistent with previous findings (10–13) suggesting that because threats attract and hold attention (they are harder to disengage), their presence as task-irrelevant distractors results in reduced performance on the primary task. Finding the first number is essentially based on attentional orienting that relies more on bottom-up than top-down processing (21, 52). The presence of BII threats overrides task instructions, quickly captures attention, makes disengagement more difficult, and consequently results in slower RTs. In contrast, snakes did not show such an effect, and RTs were faster in the snake condition compared to the BII condition. This suggests that snakes did not have an effect on involuntary attention or that participants were able to suppress it, thus negating its interference. While previous studies (73, 74) have shown that the attentional signal produced by a visually salient stimulus in the visual field can be actively suppressed by the observer prior to attentional capture, this effect has not been shown for threat-related stimuli. Furthermore, previous studies (38, 75, 76) showing that shapes associated with various threats are more salient than other visual features often used snake-related visual cues such as curvilinear shapes (similar to the body of a snake) or downward pointing Vs (geometrically similar to the head of a snake). However, more recent studies (77–80) have begun to question the validity of these studies due to methodological issues, leading to the impression that the appearance of a snake effect depends on the paradigm used and is less universal than previously thought. Nevertheless, our results show that different types of threat can have distinct effects on attentional orienting.

We found evidence for the arousal stimulation effect only for BII-related stimuli in terms of overall search performance. BII-related images distracted participants, resulting in slower RTs in the medium arousal condition, but this effect was overcome by the arousal difference in the high arousal condition. Overall search performance is based on executive attention related processes, including attentional control and allocation of working memory resources (21, 52). Highly threatening images might improve the efficiency of executive control (81), leading to a more efficient allocation of available resources (seemingly increasing working memory capacity), and thus to improved task performance (82). Interestingly, we again found no evidence that snake stimuli had any effect on task performance. Since performance was better on snake trials compared to BII trials (and did not differ from neutral trials), we might assume that they did not interfere with the primary task. Whether this lack of interference was due to successful inhibition or other factors will require future studies using physiological measures or eye-tracking.

The results of our exploratory analysis of personality traits suggest further differences between the two types of threat used in our study. The only overlapping feature was that people with higher levels of trait anxiety performed worse in terms of overall search performance than those with lower levels of anxiety. This is consistent with our hypothesis and with the results of previous studies showing that threats are more likely to distract those with high (as opposed to low) levels of trait anxiety (41, 42). Furthermore, anxiety may lead to a disruption of the executive control system due to the need to constantly regulate symptoms, resulting in a reduced capacity for other cognitive demands (83, 84). While disgust sensitivity had similar effects in the BII and snake conditions, improving performance, it impaired overall search for BII stimuli while finding the first number for snakes. Previous studies (30, 31, 85) have argued that the fear response is driven by disgust for phobias (and associated threats) that are better described by the disease-avoidance model (i.e., avoidance of objects that may cause disease or illness). While it has been shown that snakes can also elicit disgust (57, 86), our results suggest that this only affects involuntary attentional processes. However, for BII threats that clearly fit this model, disgust is a core emotion (87) that drives its effect on overall search performance, possibly explaining the arousal effect we found in this condition. For snakes, we also found an effect of fear, resulting in more interference and slower RTs to find the first number. Thus, again, it seems that snakes may only affect attentional orienting, but not executive processes. Further studies are needed to disentangle the unique effects of disgust and fear with respect to snakes. These findings may also be relevant to the motivational relevance account (45, 46), which posits that stimuli with heightened personal or biological significance are more likely to attract attention. The differential effects observed between snake and BII threats, as well as the distinct roles of fear and disgust, are consistent with the idea that attentional biases are driven not only by fear responses but also by the motivational salience of stimuli, such as their relevance to survival or disease avoidance. Further exploration of this framework may provide additional insights into the observed patterns of attentional engagement and executive interference. Finally, in the BII condition, the use of adaptive emotion regulation strategies led to better performance in finding the number one. Further, our result could suggest that the use of adaptive emotion regulation strategies may become reflexive for those who are more prone to use them. This fits well with the notion that adaptive emotion regulation strategies may help to overcome phobic fear (28, 47).

Some limitations of the study should be noted. First, we had a relatively small sample size for examining individual differences. Although we conducted a power analysis that confirmed that our study was well-powered for the behavioral variables central to our primary focus, the smaller sample size may limit the generalizability of findings related to individual differences. Second, the study may not have included enough participants with high levels of snake or blood-injection-injury specific fear to fully explore these effects. However, our goal was to approach these variables dimensionally rather than categorically. This approach is consistent with our hypothesis that the influence of fear on attentional mechanisms is proportional to its level, suggesting a linear relationship. These exploratory findings are promising and provide a foundation for future research. Third, our paradigm introduced an element of uncertainty in that the images could appear in one of four randomly selected locations. Uncertainty is known to be associated with anxiety, and evidence suggests that individual differences in intolerance of uncertainty (IU) may interact with attentional processes (88). Although we measured trait anxiety in our study, we did not include specific measures of IU. It is possible that IU could influence the relationship between anxiety and attentional processes observed in our study.

In sum, our study contributes to a deeper understanding of how various threat types affect attentional processes. Specific threat types may elicit distinct behavioral responses, highlighting the importance of considering the underlying mechanisms of different threats in attention research. Our findings underscore the importance of considering individual differences in anxiety, disgust sensitivity, and emotion regulation strategies in threat-related research. Further work is needed to elucidate the unique effects of fear and disgust on attentional mechanisms and to explore potential interventions for managing phobic responses.

## Data Availability

The datasets presented in this study can be found in online repositories. The names of the repository/repositories and accession number(s) can be found below: https://osf.io/a62z7/.
